# Effects of Antagonists on Mycotoxins of Seedborne *Fusarium* spp. in Sweet Corn

**DOI:** 10.3390/toxins11080438

**Published:** 2019-07-25

**Authors:** Mary E. Ridout, Bruce Godfrey, George Newcombe

**Affiliations:** 1 University of Idaho Extension, Washington County, College of Agriculture and life Sciences, Weiser, ID 83672, USA; 2 Department of Civil & Environmental Engineering, University of Washington, Seattle, WA 98105, USA; 3 Department of Forest, Rangelands and Fire Sciences, University of Idaho, Moscow, ID 83844, USA

**Keywords:** *Fusarium verticillioides*, *Fusarium proliferatum*, fumonisin, deoxynivalenol, fungal antagonists

## Abstract

*Fusarium* species coexist as toxigenic, systemic pathogens in sweet corn seed production in southwestern Idaho, USA. We hypothesized that fungal antagonists of seedborne *Fusarium* would differentially alter production of *Fusarium* mycotoxins directly and/or systemically. We challenged the *Fusarium* complex by in vitro antagonism trials and in situ silk and seed inoculations with fungal antagonists. Fungal antagonists reduced growth and sporulation of *Fusarium* species in vitro from 40.5% to as much as 100%. *Pichia membranifaciens* and *Penicillium griseolum* reduced fumonisin production by *F. verticillioides* by 73% and 49%, respectively, while *P. membranifaciens* and a novel *Penicillium* sp. (WPT) reduced fumonisins by *F. proliferatum* 56% and 78%, respectively. In situ, pre-planting inoculation of seeds with *Penicillium* WPT systemically increased fumonisins in the resulting crop. *Morchella snyderi* applied to silks of an F1 cross systemically reduced deoxynivalenol by 47% in mature seeds of the F2. Antagonists failed to suppress *Fusarium* in mature kernels following silk inoculations, although the ratio of *F. verticillioides* to total *Fusarium* double with some inoculants. *Fusarium* mycotoxin concentrations in sweet corn seed change systemically, as well as locally, in response to the presence of fungal antagonists, although in *Fusarium* presence in situ was not changed.

## 1. Introduction

Fungi produce many biochemicals through metabolic processes and in response to stimuli [1]. In fact, many fungi in the plant microbiome engage in “biochemical warfare” among themselves and with other microbes in the community [2]. They produce numerous defensive compounds to compete against other microbes and defend their space and resources [3]. Many of the compounds have been exploited by humans for antibiotic activity [4]; others pose a threat as “mycotoxins”, metabolites with toxic effects on mammals [5].

*Fusarium verticillioides*, *F. proliferatum*, and *F. graminearum* are major maize pathogens and produce several mycotoxins [6]. *Fusarium poae* is a common weak pathogen in maize but also produces a number of toxic metabolites [7]. The former two produce fumonisins, while the latter two produce deoxynivalenol (DON). Both toxins are regulated by the US Food and Drug Administration [8,9] and may travel systemically throughout the plant beyond the *Fusarium*-infected tissues [10,11]. Although neither mycotoxin group is necessary to the *Fusarium* species for infection and disease development in maize [12], their production has been correlated with increased severity of disease [13,14] and genes for pathogenicity have been found to be linked to mycotoxin biosynthesis [15,16,17]. These metabolites also have been observed to interact with host resistance genes [18] and facilitate infection of the host [12,13]. However, production of toxins can be specific to the pathogen strain, not just species, [7,19,20], a behavior frequently obfuscating the exact functions of these biochemicals [21].

Other microbes are known to antagonize or interact with *Fusarium* in the ear microbiome and root systems of maize [22,23,24]. Such microbial competition can effect changes in *Fusarium* mycotoxin production [25,26,27,28,29]. In fact, Keyser et al. [30] postulated that fumonisins could function as defense compounds when they found that fumonisin extracts reduced growth of several *Fusarium* competitors from the maize ear microbiome. Bacon et al. [26,31] similarly found that *F. verticilliodes* produced fusaric acid toxic to the *Fusarium* biocontrol agent *Bacillus mojavensis* when challenged with the bacterium. Conversely, Yates et al. [32] found that *Trichoderma viride* could reduce fumonisin production by *F. verticillioides*. Similarly, Chatterjee et al. [33] found that *Clonostachys rosea* inhibited fumonisin synthesis by *F. verticillioides* but did not degrade the toxin. Hebbar et al. [34] identified a number of bacteria, including several *Pseudomonas* species, in maize plants producing antifungal metabolites inhibitory to *Fusarium verticillioides*. In other cases, effects were more complex [32].

*Fusarium* species themselves interact chemically within the complex. More than one *Fusarium* species may colonize a host [35] and some, like *Fusarium verticillioides*, may colonize the host asymptomatically [36,37]—all producing a variety of toxins. For instance, *Fusarium graminearum* and *F. verticillioides* are known to interact significantly both in laboratory antagonism assays and in the field in terms of both growth and toxin production [35,38,39]. However, the direction of the interaction (antagonistic or synergistic) is highly dependent on environmental parameters [21,38,39].

The southern region of Idaho, USA produces 70% of the world’s sweet corn (*Zea mays* convar. *saccharata* var. *rugosa*) seed [40]. Here in the semi-arid, continental climate and alkaline soils [41,42], *Fusarium verticillioides* and *F. proliferatum* co-occur in the *Fusarium* pathogen complex in sweet corn seed production [43,44]. Seed becomes infected via wind-driven and rain-splashed spores colonizing ears and re-infects successive crops systemically from infected seed surfaces and soil [45,46]. In addition to multiple *Fusarium* species, numerous other microbes colonize the seeds through the silks, some of which are antagonistic to the *Fusarium* species

Most frequently, attempts to understand the interactions of other microbes with toxigenic *Fusarium* species examine the interaction of a single antagonist with a single *Fusarium* species [24,27,28,47]. Yet, the complexity of the ear microbiome in maize [22,23,27] indicates the biochemical behaviors may be much more complex as well. In a series of field trials, we surveyed the *Fusarium* pathogens in sweet corn fields in Nampa, ID, USA.

We hypothesized that different *Fusarium* species present during infection of the ears at silking would differentially alter mycotoxin production in response to antagonism by five fungal species with putative antagonism toward *Fusarium* species First, we confirmed antagonism of *F. verticillioides* and *F. proliferatum* by our putative antagonists and tested *Fusarium* isolates for fumonisin and DON production. To test our hypothesis, we quantified effects of fungal antagonists on sporulation of *F. verticillioides* and *F. proliferatum* and measured fumonisin production of both *Fusarium* when antagonized. We hypothesized that antagonists applied to ears at silking would induce changes in *Fusarium* mycotoxin production in situ, and that effects would occur locally in treated ears and systemically in plants grown from treated seed. We tested this hypothesis in 2015 field trials with the same suite of fungal antagonists. We inoculated emerging silks at flowering and seed at planting to compare local and systemic effects on mycotoxin content. To confirm systemic effects, we conducted a second year of field trials in 2016 and planted seeds developed from 2015 silk inoculations trials. Finally, we used quantitative polymerase chain reaction (qPCR) on mature seed from 2015 silk inoculation trials to determine the effects of fungal antagonists on *Fusarium* populations in the seed.

## 2. Results

### 2.1. Fusarium Species Present in Sweet Corn Seed from Nampa, Idaho, USA

**Figure 1:**
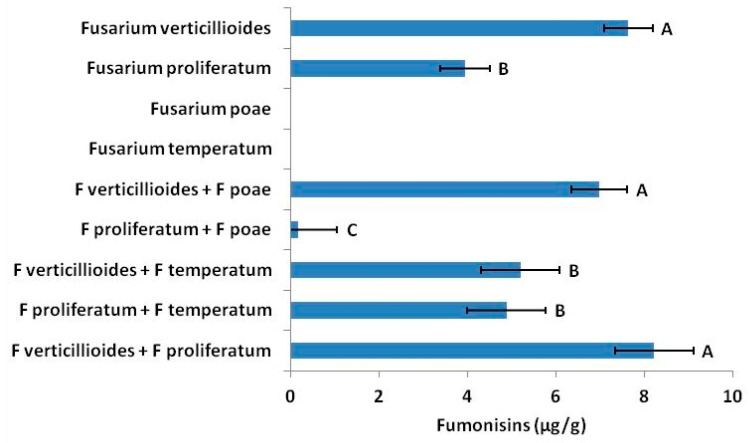
Co-occurring *Fusarium* species also altered fumonisin production of *F. verticillioides* and *F. proliferatum* in vitro. Although neither produced fumonisin, *F. poae* reduced fumonisin production by *F. proliferatum* by 96%, and *F. temperatum*, reduced fumonisin by *F. verticillioides* by 32%. Error bars indicate standard error (*α* = 0.05).

Four major *Fusarium* species were identified from the ears of sweet corn seed fields of Nampa, ID, USA: *Fusarium verticillioides*, *F. proliferatum*, *F. temperatum* [48] and *F. poae*. *Fusarium proliferatum* and *F. verticillioides* were most abundant in ears from 2014 and 2015 field trials (>95% isolation frequency in non-sterilized seed). *Fusarium temperatum* occurred at relatively low frequency (<30%) in both years. Only three isolates of *F. poae* were observed and only one was successfully isolated in the 2015 seed. As expected, when analyzed alone, only *F. verticillioides* and *F. proliferatum* produced detectible fumonisins. None of the isolates produced DON (data not shown). *Fusarium proliferatum* averaged 4 mg of fumonisins per kilogram of fungal and substrate material whereas *F. verticillioides* averaged nearly twice that amount (*p* < 0.0001, Figure 1).

### 2.2. Antagonists of Sweet Corn Fusarium Species

**Figure 2:**
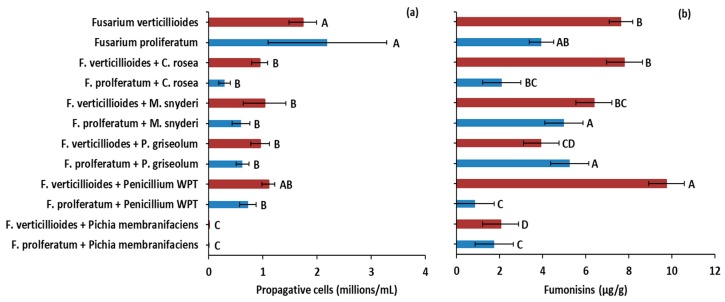
(**a**) Fungal antagonists of *Fusarium* species strongly reduced growth and sporulation of *F. proliferatum* and *F. verticillioides* in antagonism assays. Filamentous fungi antagonized *F. proliferatum* to a greater extent (67% to 87% reductions in sporulation) than *F. verticillioides* (37% to 46% reductions in sporulation). The yeast *P. membranifaciens* prevented development of both *Fusarium* species in broth culture. (**b**) However, antagonism had varying effects of fumonisin production that were specific to *Fusarium* sp. Both fumonisin producers antagonized by *P. membranifaciens* produced approximately 50% less fumonisin. When challenged with *Penicillium* WPT, *F. proliferatum* produced 78% less fumonisin while *F. verticillioides* produced 28% more. Antagonism with *P. griseolum* reduced fumonisin in *F. verticillioides* by 73% but had no effect on *F. proliferatum*. Error bars indicate standard error (*α* = 0.05). Letters above red or blue bars indicate significant differences within a treatment set (*F. verticillioides*, red; *F. proliferatum*, blue).

In laboratory antagonism assays, four fungal species isolated from native plants in Idaho, USA were found to strongly antagonize *F. verticillioides* and *F. proliferatum*. *Penicillium* sp. nov. WPT (hereafter *Penicillium* WPT), *Penicillium griseolum*, *Clonostachys rosea*, and *Morchella snyderi* inhibited growth and sporulation of both *F. verticillioides* and *F. proliferatum*. *Clonostachys rosea* also overgrew *Fusarium* species in vitro and sporulated on the surface of the parasitized *Fusarium* mycelia. Our strain of *Pichia membranifaciens*, which we isolated from immature sweet corn kernels from the field in 2014, inhibited growth of *F. verticillioides* and *F. proliferatum* in vitro as long as it remained viable. In broth culture, *P. membranifaciens* reduced the reproductive capacity of both *F. proliferatum* and *F. verticillioides* by suppressing conidia production by 99.9% compared to *Fusarium*-only controls (Figure 2a). *Penicillium* WPT, *P. griseolum*, *M. snyderi*, and *C. rosea* reduced sporulation of *F. proliferatum* by 67%, 71%, 73%, and 87% (*p* < 0.01), respectively (Figure 2a). *Fusarium verticillioides* was only slightly more resistant to filamentous antagonists with *M. snyderi*, *P. griseolum*, and *C. rosea* reducing sporulation by 40.5%, 45%, and 46%, respectively (*p* < 0.05). However, *Penicillium* WPT failed to reduce sporulation of *F. verticillioides* (*p* > 0.05) (Figure 2a).

### 2.3. Antagonists Alter Fumonisin Production In Vitro

In vitro, *Fusarium* antagonists significantly altered fumonisin production by *F. verticillioides* and *F. proliferatum* (Figure 2b). *Fusarium verticillioides* antagonized by *P. griseolum* and *P. membranifaciens* produced 49% (*p* = 0.0003) and 73% (*p* < 0.0001) less fumonisin, respectively, than it produced without antagonist pressure. *Fusarium proliferatum* challenged with *P. membranifaciens* and *Penicillium* WPT produced 56% (*p* = 0.0396) and 78% (*p* = 0.0040) less fumonisin, respectively, than when unchallenged. By contrast, when *F. verticillioides* was challenged with *Penicillium* WPT, the pathogen increased fumonisin production by nearly 28% (*p* = 0.0359).

Although antagonists were effective at altering fumonisin expression by *Fusarium* species, co-occurring *Fusarium* species were equally effective (Figure 1). When *F. proliferatum* was challenged with co-occurring *F. poae*, it produced 96% (*p* = 0.0005) less fumonisin than when unchallenged, and antagonism by *F. temperatum* reduced fumonisin production of *F. verticillioides* by 32% (*p* = 0.0213).

### 2.4. In Situ Antagonism is not Predicted In Vitro

**Figure 3:**
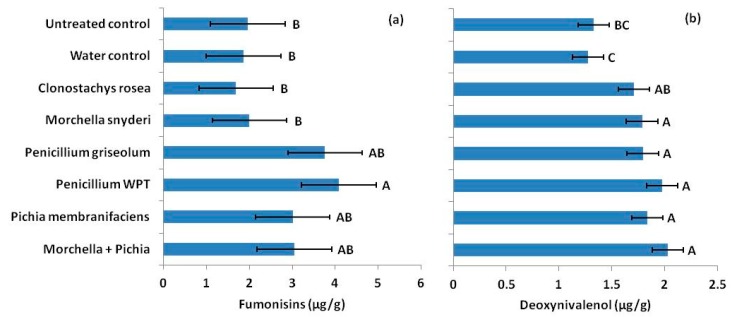
(**a**) In contrast to laboratory antagonism assays, fungal antagonists applied to developing ears at silking, mostly increased fumonisin concentrations in mature seed. In fact, *Penicillium* WPT nearly doubled fumonisins in treated ears compared to ears receiving no treatment (dry control). (**b**) Application of fungal antagonists also increased DON concentrations in mature seed compared to ears receiving no treatment (dry control) or ears that just receive distilled water application. Error bars indicate standard error (*α* = 0.05).

In vitro antagonism failed to predict effects in the field. In the 2015 silk inoculation trial, fumonisins were not reduced in ears treated with putative antagonists (Figure 3a). In fact, ears treated with *Penicillium* WPT at silking had nearly twice the fumonisin content of untreated ears from the control (*p* = 0.0026). DON production was low. However, treatments effects were still detected. DON was significantly higher (*p* = 0.0037) in seed from ears treated with most antagonists at silking, from a 34% increase in seed inoculated with *M. snyderi* to 52% in seed treated with *M. snyderi* and *P. membranifaciens* combined (Figure 3b). Only *C. rosea* failed to increase DON in mature seed compared to untreated ears (*p* = 0.0737, *α* = 0.05).

### 2.5. Putative Antagonists Act Systemically In Situ

**Figure 4:**
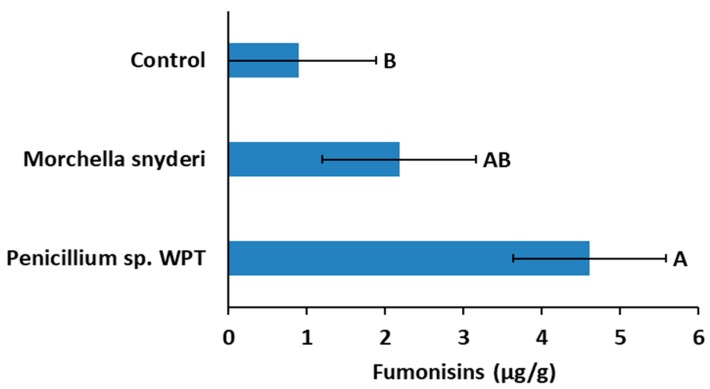
Applications of *Penicillium* WPT to seed at planting resulted in significant increases in fumonisin concentrations in mature seed. Application of *Morchella snyderi* slightly but not significantly increased fumonisin concentration in seed. Fumonisins can be systemically transported in plant solution. Error bars indicate standard error (*α* = 0.05).

Effects of antagonists on mycotoxin production in sweet corn ears were also found to be systemic. When seeds were treated with antagonists at planting in the 2015 field trial, seed treatment with *Penicillium* WPT resulted in a five-fold increase in fumonisins (*p* = 0.0217, Figure 4) compared to untreated controls but had no effect on DON (*p* > 0.05, data not shown) in mature seed from that planting.

**Figure 5:**
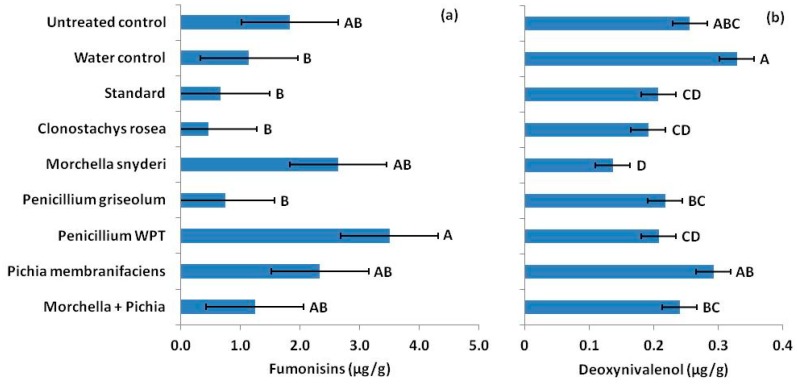
Antagonists applied emerging silks in an F1 hybrid increase in 2015 stimulated carry-over effects in the susceptible F1 hybrid the following year (2016). (**a**) Although average fumonisin content was both increased and reduced among treatments, differences were not significant. (**b**) However, seed from the *Morchella snyderi* treatment had half the DON concentrations of the untreated (dry) control. Error bars indicate standard error (*α* = 0.05).

This ability to alter production of mycotoxins systemically was confirmed to be long-term in carry-over effects in the 2016 field trial. Mycotoxin concentrations in ears of plants grown from seeds from the 2015 silk inoculation trial were highly variable compared to concentrations in the 2015 seed (Figure 5), but effects of antagonists were observed to carry over to the second generation. Plants grown from seeds inoculated at silk with *M. snyderi* had 47% less DON (*p* = 0.0025) than plants grown from untreated seed (Figure 5b).

### 2.6. Putative Antagonists and Fusarium Colonization of Seed In Situ

**Figure 6:**
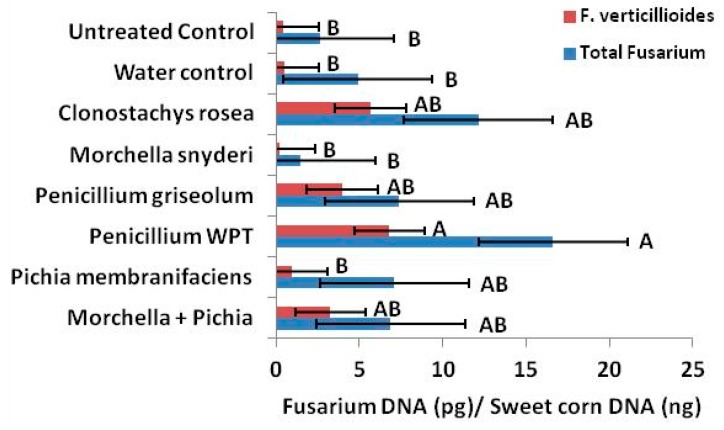
Quantitative PCR mostly showed increases in *Fusarium* colonization with the addition of fungal inocula applied to developing ears via emerging silks. Inoculation of silk with *Penicillium* WPT strongly increased both total *Fusarium* and *F. verticillioides* presence. *Morchella snyderi* was the only silk inoculation treatment that reduced colonization of both *F. verticillioides* and total *Fusarium* but not significantly. Error bars indicate standard error (*α* = 0.05). Letters above red or blue bars indicate significant differences within a treatment set (*F. verticillioides*, red; Total *Fusarium*, blue).

**Figure 7:**
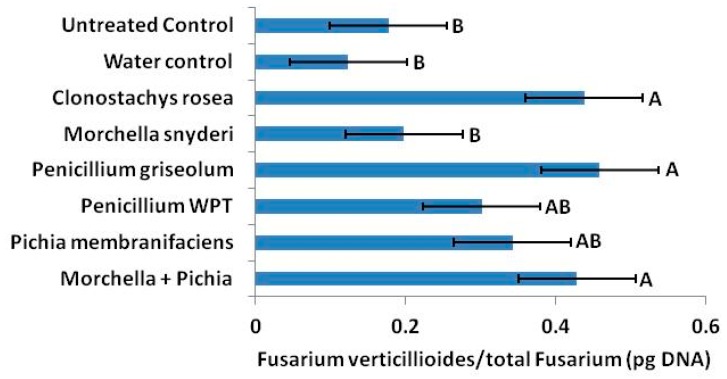
Although concentrations of *Fusarium* in mature seed were not significantly influenced, applications of *C. rosea*, *P. griseolum*, and *Morchella* + *Pichia* had a higher average ratio of *F. verticillioides* to total *Fusarium* than the untreated (dry) control (*p* < 0.05). Error bars indicate standard error (*α* = 0.05).

Putative antagonists strongly altered mycotoxin production by *Fusarium* species locally, systemically, and across generations in the field, and effects on the colonization of mature kernels were also observed by qPCR. However, antagonism did not result in reductions in seedborne *Fusarium*. In fact, seed treated at silking with *Penicillium* WPT had over 6 times more total *Fusarium* DNA to seed DNA than untreated controls and 15 times more *F. verticillioides* (Figure 6). The abundance of *F. verticillioides* relative to total *Fusarium* was unaffected by *Penicillium* WPT. By contrast, *C. rosea*, *P. griseolum* and the combination treatment of *Morchella* and *Pichia* more than doubled the ratio of *F. verticillioides* to total *Fusarium* (Figure 7).

## 3. Discussion

When challenged with fungal antagonists, *Fusarium* species from sweet corn seed fields in S. Idaho, USA, responded with detectable changes in mycotoxin production even while having no effect on the overall populations of the pathogens in seed. Not only were mycotoxin concentrations changed by direct antagonistic interactions in vitro but were altered by inoculations of antagonists locally in the ear. Moreover, inoculation with the novel *Penicillium* sp. WPT and *Morchella snyderi* effected systemic changes in accumulation of those fumonisins and DON in the seed.

Fumonisins [10] and DON [11] are known to move systemically in plants, concentrating in tissues without colonization of those tissues by their fungal producers. That single microbes applied either to the seed at planting (Figure 4) or to developing ears in silk (Figure 5) could alter those concentrations in first- and second-generation seed lots (respectively) in a complex abiotic and biotic environment would indicate that far more than the *Fusarium* species themselves are involved in mycotoxin accumulation in cereals. Whether these toxins are produced by *Fusarium* species as defense compounds cannot be determine by our study. However, challenging *Fusarium* species with antagonists did promote changes in both fumonisin and DON concentrations relative to unchallenged *Fusarium* both in vitro and in situ, indicating that other microbes in the ears of sweet corn or maize may strongly influence bioaccumulation of these toxins.

The novel species *Penicillium* sp. isolate WPT mostly stimulated fumonisin production by *Fusarium* both in vitro and in situ. The strongest effects of inoculation with the antagonist were systemic in the 2015 field trial in which seed was treated prior to planting. Mature seed produced on plants that developed from treated seed averaged five times the fumonisin content of seed from untreated control plants. It is interesting to note that *Penicillium* WPT produces griseofulvin (J. David Miller, Carlton U. ON CA, personal communication), an anti-fungal used pharmaceutically and known to suppress fungal disease in crops [49,50]. Griseofulvin has been shown to move systemically in plants [51,52], including major grass crops such as wheat, even from soil-borne microbes [53].

In vitro, antagonism by *Penicillium* WPT stimulated fumonisin production by *F. verticillioides* but reduced production by *F. proliferatum*. *Penicillium griseolum* had almost the opposite effects: it reduced fumonisin output by *F. verticillioides* while having no effect on *F. proliferatum* fumonisins. *Penicillium griseolum* had no effect either locally or systemically in situ, despite producing low concentrations of griseofulvin (J. David Miller, Carlton U. ON CA, personal communication), indicating that microbial biochemical interactions are likely specific to species. Griseofulvin accumulation was not determined either in vitro or in situ, so that no conclusions may be drawn as to its effects against *Fusarium* species from sweet corn and consequent effects on fumonisins. However, any biochemical antagonism between these *Penicillia* and *Fusaria* species would appear to be highly species dependent.

Just as antagonistic interactions were mostly species dependent, responses were also context dependent. Even the effects of *Penicillium* WPT antagonism on systemic fumonisin accumulation were weaker in the 2016 trials compared to 2015 trials. Whether the seed was inoculated at planting or the antagonist was introduced into the seed via the silks during ear development led to different systemic effects from *M. snyderi* on DON accumulation in seed. Treatment of seeds at planting with *M. snyderi* had no effect on DON in the subsequent crop. However, inoculation of developing ears at silking with *M. snyderi* reduced DON accumulation to half that of the untreated control in second-generation seed produced from that sown in 2016. By contrast, *Fusarium* responded to *M. snyderi* inoculation in developing ears by increasing DON concentrations in the seed. Different species within the genus have been known to produce anti-microbial compounds [54,55,56], but little is known about role of context in their efficacy. There is no record of anti-microbial activity for *M. snyderi*, but it both reduced conidial development by *F. verticilioides* and *F. proliferatum* in vitro and triggered reduced DON concentrations in field seed by systemic mechanisms.

*Clonostachys rosea* was isolated from immature kernels following silk inoculations, but we did not analyze either seed or PCR products to determine the persistence of fungal inoculants in samples tested for mycotoxins and *Fusarium* abundance. Although inoculations of *Fusarium* antagonists to seed and ears resulted in differing mycotoxin accumulations in subsequent seed, this response does not indicate a persistence of the inoculants within the ear microbiome. Effects could have been the result of more complicated interactions than direct antagonism between inoculants and target *Fusarium* species. Moreover, effects of antagonism might outlast persistence of the inoculant either form systemic biochemical accumulations (as might be possible with *Penicillium* WPT) or indirectly via changes within the existing microbial community of the developing kernel. Just as bioactive secondary metabolites like griseofulvin and the *Fusarium* mycotoxin zearalenone may persist in tissues lacking the physical presence of source microbes [51,52,53,57], changes in biochemical constituency and microbial communities (e.g., relative abundance) may result from initial inoculations and persist even while the inoculant.

The consequences of the complexity of the plant microbiome on seed mycotoxin concentrations cannot be underestimated. *Fusarium proliferatum* and *F. verticillioides* responded very differently to the same antagonists in controlled pairings. In situ, interactions with antagonists are no longer pairings but are subject to multiple species interactions. We isolated four *Fusarium* species from seed, immature kernels, and vegetative tissues from the field. Our findings were consistent with findings by Mohan and Wilson [58] with *F. verticillioides* being most common [48] followed by *F. proliferatum*. *Fusarium temperatum* and *F. poae* were isolated, but rare. However, none of our isolates produced DON in vitro, yet DON accumulated in mature seed and concentrations varied with antagonist interactions. It is likely that other *Fusarium* species are present in the system that were not successfully isolated. For example, *F. graminearum* has been repeated isolated from wheat heads in S. Idaho [59]. *Fusarium* species are known to antagonize each other [35,38,39]. Moreover, genetic variation among strains of the same *Fusarium* sp. vary in mycotoxin production [20]. From our own data we saw that interactions among *Fusarium* species (Figure 1) changed fumonisin concentrations. Although only one or two species may produce a particular mycotoxin in situ, other *Fusarium* present may influence accumulation and concentrations in seed.

In addition to multiple *Fusarium* species, *Penicillium, Aspergillus, Rhizopus, Cladosporium* and *Trichoderma* species are known to be present in sweet corn seed [44,60,61]. We obtained our isolate of *Pichia membranifaciens* as well as several unidentified bacteria and fungi from seed produced in 2014 field trials. In vitro, *P. membranifaciens* effectively suppressed both growth and fumonisin production by *F. proliferatum* and *F. verticillioides*. What effects these natural populations of antagonists might have as “third parties” to inocula can be partially seen in our 2016 field trials (Figure 5). Although *M. snyderi* alone reduced DON output to nearly half that in plants from untreated seed, concentrations in plants in the dual *M. snyderi* + *P. membranifaciens* inoculation treatment was similar to untreated. Thus, other microbes besides inocula play no small role in *Fusarium* output of mycotoxins.

Antagonistic inocula and perhaps naturally occurring antagonists are also likely to influence relative mycotoxin concentrations in seed by selectively antagonistic behavior toward *Fusarium* species. In our qPCR analysis, we determine that *C. rosea*, *P. griseolum* and the combination treatment *M. snyderi* + *P. membranifaciens* increased the ratio of *F. verticillioides* to total *Fusarium* (Figure 7).

Environmental effects, such as temperature and relative humidity, are known to contribute to variation in mycotoxin production [21,38,62,63]. To follow commercial rotation schedules, field sites changed each year of our study. We saw overall DON and fumonisin concentrations decline in 2016 compared to 2015, in the case of DON by an order of magnitude. This may have been in part due to changes in site location or the result of differences in precipitation and temperature patterns between the two years. Reductions in 2016 may also have resulted from shifts in species relative abundance of microbiota, specifically the Fusarium complex, within the ears.

Interactions in situ were mostly not predicted by in vitro results. Even where strong, antagonistic suppression of both fumonisin production and conidial development were observed in laboratory experiments, antagonists were mostly not suppressive in situ. For example, all antagonists strongly reduced conidial development of *F. verticillioides* in vitro; in situ, seed treated with *Penicillium* WPT at silking had over 15 times more *F. verticillioides* than untreated seed. Although in vitro interactions are frequently used to screen for microbial antagonism they often fail to have predictive value for field or in situ response variables [64,65,66].

If we would truly understand the variables that contribute to *Fusarium* mycotoxins in sweet corn seed or maize production, we must begin to study the system as a whole in situ, where effects of the biotic environment as well as the abiotic might be studied and dissected. If antagonistic inocula can have systemic effects on fumonisin and DON concentrations in sweet corn seed in the field, it is likely that we have underestimated the effects of the entire plant microbiome and microbial “biochemical warfare” [2] on mycotoxins.

## 4. Materials and Methods

### 4.1. Isolating the Sweet Corn Fusarium Complex

*Fusarium* species were isolated from seed samples collected from sweet corn field trials in Nampa ID, USA during 2014 and 2015 field trials. Seed samples were collected when kernels were immature and again at maturity. Seeds were plated onto 4% potato dextrose agar (PDA, Difco Laboratories, Inc., Detroit, MI, USA) and incubated for up to four weeks at 22 °C. *Fusarium* isolates were aseptically transferred to pure culture on 4% PDA.

### 4.2. Fusarium Species Identification

Twelve isolates of *Fusarium* species were selected for sequence identification to represent individual morphotypes from the pure culture collection. Genomic DNA from the 12 cultured *Fusarium* isolates was amplified using the ITS-1F and LR3 and the EF1 and EF2 primer pairs. PCR products were checked by agarose gel electrophoresis and purified by PEG/NaCl precipitation. These templates were sequenced with BIgDye3.1 (Applied Biosystems®, Foster, CA, USA) using the same primers used for amplification and analyzed on an ABI3130XL Genetic Analyzer (Life Technologies, Inc., Thermo Fisher Scientific, Waltham, MA, USA) with an 80cm capillary array. Forward and reverse read consensus sequences were checked against GenBank and candidate *Fusarium* species were identified from the EF1/2 sequences at 99–100% sequence identity and cross-validated by micromorphology.

### 4.3. Selecting Fungal Antagonists

Fungal antagonists of *Fusarium* species were selected for laboratory and field research using in vitro antagonism assays. Fungi of interest were obtained from indigenous plant microbiomes of the intermountain west, including *Pseudotsuga menziesii* var. *glauca*, *Abies grandis*, and *Pinus ponderosa* [67]. These fungi were previously observed to antagonize *F. proliferatum* isolated from *P. menziesii* in culture. A well-known mycoparasitic fungus *Clonostachys rosea*, isolated from roots of *P. menziesii* var. *glauca* was deliberately selected for its mycoparasistic activity, and a fungistatic yeast, *Pichia membranifaciens* isolated from sweet corn was also selected. New in vitro assays were designed to screen for antagonistic behavior by inoculating petri plates of 4% PDA with paired antagonists. A center line was drawn across the diameter of the plate. *Fusarium* species were inoculated into the center of the space on one side of the plate approximately 1 cm from the line. Directly opposite, one antagonist was inoculated onto the plate 1 cm from the line. Control plates were grown with only the *Fusarium* or an antagonist. Observations were taken of the plates for five to seven days at 22 °C. Fungi that appeared to reduce growth and spread of *Fusarium* species across the center line of the plate and/or appeared to reduce growth or stimulate pigmented secondary metabolites with physical contact were subjected to a quantitative test in broth antagonism cultures.

### 4.4. In Vitro Quantitative Tests of Antagonism

*Fusarium* species were co-inoculated with selected antagonists in paired antagonism broth cultures with a *Fusarium* culture broth containing a solution of 2% sucrose, 2% glucose, 1.5% peptone, and 1% potato dextrose adjusted to a pH of 6.2. Each 50-mL culture contained either *F. verticillioides* or *F. proliferatum* and a single antagonist. Five antagonists observed to antagonize *Fusarium* species in vitro were tested: *Clonostachys rosea*, *Morchella snyderi*, *Penicillium* sp. nov. WPT, *Penicillium griseolum*, and *P. membranifaciens*. Control cultures contained only *Fusarium* species

To inoculate the broth cultures, two-week-old PDA plate cultures of each fungus (antagonists or *Fusarium* spp.) were flooded with sterile distilled water (SDW) and scraped with a bent, glass rod to loosen fungal propagules into solution. Propagules of *M. snyderi*, which forms heavy hyphal filaments and sclerotia on PDA, were loosened using a scalpel blade. One hundred microliters of *Fusarium* propagule solution were inoculated into each 100 mL test tube containing 50 mL of the broth solution for each antagonism. For each antagonism treatment, 100 µL of antagonist propagule solution were added. Four replicate cultures were made for each treatment with four antagonist-free *Fusarium* cultures for a control. *Fusarium proliferatum* and *F. verticillioides* solutions were inoculated into their respective broth cultures at 10^7^ propagules per ml. Starting cultures contained 2 × 10^5^ propagules per ml of each pathogen. Inocula were not standardized among antagonists since effects among antagonists were not comparable due to highly different mechanisms of antagonism and the antagonists were not being compared. Starting concentrations in propagules per ml of broth were as follows: *C. rosea*, 7 × 10^4^; *M. snyderi*, 7 × 10^1^; *P. griseolum*, 3.8 × 10^4^; *Penicillium* WPT, 3.4 × 10^5^; *P. membranifaciens*, 1.2 × 10^4^.

Following inoculation, the broth cultures were incubated for three days at 22 °C on a shaker at a constant 150 rpm. On day three, counts of *Fusarium* conidia were made in each culture using a Neubauer cell counting chamber. Counts were computed to the concentration of conidia per ml.

### 4.5. Mycotoxin ELISA—Direct Antagonisms

Sequenced *Fusarium* isolates from the sweet corn seed isolate collection were assayed for their ability to produce mycotoxins. Three putative fumonisin producers and a single putative deoxynivalenol (DON) producer were assayed. Pure cultures of the four isolates were cultured on 4% PDA for ten days at 22 °C. Ten grams of culture material (mycelium and agar) were removed from the plates and placed in jars. Fumonisins were extracted with 70% HPLC-grade methanol at a ratio of 1:5 (10 g culture material: 50 mL methanol). DON extracts were made with deionized (DI) water at a 1:5 ratio (culture material: DI water). Extracts were then analyzed using ELISA 96-well mycotoxin enzyme kits (Agriquant^®^, Romer Labs Inc., Newark, DE, USA) according to manufacturer’s instructions. ELISA plates were scanned using a microplate spectrophotometer (Powerwave^™^, Bio-Tek Instruments, Winooski, VT, USA) to obtain optical density (OD) readings. Data were transformed using a standard curve to obtain concentrations (µg/g).

Effects of antagonisms on mycotoxin production were determined with a series of in vitro assays pairing *Fusarium* species with antagonists. Five antagonists were paired with two fumonisin producers: *C. rosea*, *M. snyderi*, *Penicillium* sp. nov. WPT, *P. griseolum*, and *P. membranifaciens*. The two fumonisin-producing *Fusarium* species were also paired with the other two sequenced *Fusarium* species to determine potential mycotoxin interactions among the isolates. Fumonisin producers were inoculated onto 4% PDA plates 1 cm from the center of the plates. On the opposing side of each plate, also 1 cm from the center of the plate, one of the antagonists or co-occurring *Fusarium* isolates was plated. Eight plates were made for each of the pairings with co-occurring *Fusarium* isolates or fungal antagonists, respectively. The positive control plates consisted of the fumonisin producers without competition in the plates. Negative controls were generated for each of the five antagonists by plating each antagonist alone. Only four plates were made for each negative control. After three weeks, a 2-cm wide by 9 cm long strip of agar and fungal material running perpendicular to the dual cultures was removed from each plate. Fumonisins were extracted by submerging each strip in 70% methanol at a ratio of 1:5 (*w*/*v*, culture material:methanol). Extracts were analyzed using the ELISA technique as previously described.

### 4.6. Testing Effects of Antagonism in Situ

#### 4.6.1. 2015. Field Trial—Silk Inoculations

The summer 2015 two in situ antagonism studies were sown in Nampa ID, USA. The first antagonism study examined effects of direct antagonisms via inoculation of emerging silks of a susceptible inbred line (L2) on mycotoxin accumulation in and *Fusarium* colonization of developing seed. The study also functioned as a seed increase to generate antagonist-carrying F1 hybrid seed to be sown the following year (2016) to study potential carry-over and systemic effects from seed transmission of the inoculants to an F2 generation. The female inbred was sown in early June 2015 in a standard F1 hybrid increase design with four female rows bracketed by two male rows. The male line was sown approximately two weeks previous to planting the female line.

In late July (23 July 2015), emerging silks were inoculated with eight different antagonism treatments: *C. rosea*, *M. snyderi*, *P. griseolum*, *Penicillium* WPT, *P. membranifaciens*, *M. snyderi* + *P. membranifaciens*, a sterile water control, and an untreated control that received no wet applications. Fungal propagules (spores and macerated hyphae) were suspended in sterile distilled water at concentrations of 10^4^ propagules per ml for *M. snyderi* and 10^7^ propagules per ml for all other inocula. Inocula were not standardized among antagonists since effects among antagonists were not comparable due to highly different mechanisms of antagonism. Antagonists were applied to emerging silks using hand-held atomizers calibrated to 5 mL of suspension per ear. The *M. snyderi* + *P. membranifaciens* treatment received 2.5 mL of each antagonist for a total of 5 mL of inocula. This inoculation was repeated for three nights with inoculations beginning at approximately 6:30PM MST so that each treatment received 15 mL of suspension. Temperature and relative humidity at the silking zone were monitored over a 36-h period beginning the first evening. Treatments were arranged in an 8-block randomized block design at the head of the increase field with 10 plants treated per treatment per block. A total of 640 plants were used in the experiment.

Mature ears were harvested September 17. Ears were dried at 34 °C. Subsamples for each ear were randomly selected for quantitative PCR testing and ELISA mycotoxin assays. Later, seed from individual ears was bulked by treatment for a 2016 planting.

#### 4.6.2. 2015. Field Trial—Seed Treatment for Systemic Effects

The second trial to investigate possible systemic antagonism was planted in May 2015. Antagonist-treated seeds from the *Fusarium*-susceptible inbred L2 were sown in a replicated trial in Nampa ID. Treatments were blocked in replicated ranges of 12 rows. Each treatment was separated by a buffer row, the seeds of which were treated with standard pesticide treatments. Three antagonist treatments were tested: *M. snyderi*, *Penicillium* WTP, and a control. The antagonists were propagated on food-grade yellow cornmeal. The purpose of the cornmeal was to serve as both a culture substrate and an inoculum carrier. Inoculated cornmeal was crushed, mixed, and moistened with deionized (DI) water. The inocula were applied to seeds prior to sowing with the seeds and cornmeal carrier mixed thoroughly to coat each seed. Plain moistened cornmeal was used for the control treatment.

Starting September 25, ears were harvested on a per row basis with a 15-foot row length as a replicate. Ears were dried at 34 °C, and subsamples of mature seeds from each row were taken for mycotoxin ELISA assays.

#### 4.6.3. 2016. Field Trial—Carry-Over Effects

The F1 hybrid seeds generated from the 2015 silk inoculation trial were sown for a final field trial in May 2016. Following 2015 analyses, remaining seed was bulked by treatment. Random samples of the bulked seed were taken for sowing in the 2016 field trail. Six-row seed ranges were sown with a single treatment in a randomized block design with ranges for each treatment represented in each of four blocks. There were nine treatments sown with a total of 36 ranges. The treatments included seeds from all eight silk treatments from 2015: untreated control, water control, *C. rosea*, *M. snyderi*, *P. griseolum*, *Penicillium* WPT, *P. membranifaciens*, and seeds from the *M. snyderi* + *P. membranifaciens* inoculation. The seeds generated from the 2015 trial were hybrid seeds. They received no further treatment in 2016 prior to sowing. The ninth treatment was a subsample of the 2016 untreated control to which was applied the standard fungicide/insecticide seed treatment prior to sowing.

Ears were harvested by the 10-foot row (4 rows per range) in early October. Ears were dried in a commercial drier at a standard 34 °C. Seed subsamples of this F2 generation were taken from each row for ELISA analyses of mycotoxin content.

#### 4.6.4. Mycotoxin ELISA—2015 and 2016 Field Seed

Seeds from the 2015 field trials and the 2016 field trial were analyzed for the presence and content of both fumonisins and DON. For mycotoxin analyses of the 2015 seedborne antagonism trial, samples were bulked by row and three randomly selected rows were analyzed per treatment. Samples from the 2015 silk-inoculation field trial and the 2016 field trial were bulked by row and eight random rows from each antagonism treatment were selected for analysis. Three randomly selected samples were analyzed for both DON and fumonisins from the 2015 seed treatment field trial. Samples were ground with a coffee mill, and the mill was sanitized between samples. Fumonisins were extracted from ground samples with 70% HPLC grade methanol at a ratio of 1:5 (*w*/*v*, sample:methanol). DON was extracted with DI water at a ratio of 1:5 (*w*/*v*, sample:DI water). Mycotoxin concentrations of extracts were determine using ELISA 96-well mycotoxin enzyme kits (Agriquant^®^, Romer Labs Inc., Newark, DE, USA) according to manufacturer’s instructions. ELISA plates were scanned using a microplate spectrophotometer (Powerwave^™^, Bio-Tek Instruments, Winooski, VT, USA) to obtain OD readings. Data were transformed using a standard curve to obtain concentrations (µg/g).

### 4.7. Molecular Determination of Fusarium Antagonism

Finally, we used the quantitative polymerase chain reaction (qPCR) technique to compare quantities of *Fusarium* DNA relative to seed yield (plant DNA) from a subsample of mature seeds from the 2015 silk inoculation field trial. Only 2015 seed was analyzed due to resource constraints. Sixteen randomly selected ears were taken from each of the eight treatments, and four ears were then pooled to generate four replicates per antagonist for analysis. Thirty-gram samples of dry seeds (average 240 kernels) from each treatment and control plot were lyophilized O/N and then ground using a coffee/spice grinder (Kitchen Aid model BCG11108) which has a removable stainless-steel bowl to allow convenient cleaning between samples. Sub-samples of 100 mg of the ground seeds were ground further to a powder on a MixerMill 300 with a tungsten carbide bead in a 2ml Collection Tube (Qiagen N.V., Venlo, Limburg, Netherlands).

#### 4.7.1. Genomic DNA Extraction

DNA was extracted from the powdered seed biomass using the CTAB method [68]. The CTAB DNA preps were then subjected the PEG/NaCl precipitation. These DNAs were characterized by UV optical absorbance using a microplate spectrophotometer (Epoch^™^, BioTek Instruments, Inc., Winooski, VT, USA) and by successful endpoint PCR using tEF1 and ITS fungal primers. For greater consistency, we followed the CTAB extraction with further processing using a Qiagen DNeasy Plant kit according to the manufacturer’s instructions (Qiagen N.V., Venlo, Limburg, Netherlands) to remove residual PCR inhibitors present in the seeds. DNA quality was checked by UV optical absorbance and concentrations were measured using a Qubit^®^ fluorimeter (Invitrogen^™^, Thermo Fisher Scientific, Waltham, MA, USA) with a QuantIt^™^ broad range kit (Invitrogen^™^, Thermo Fisher Scientific, Waltham, MA, USA).

Genomic DNA was also extracted from the sequenced *Fusarium* strains isolated from plants in the field trials conducted during 2014 and 2015. *Fusarium* strains were cultured on 4% PDA plates. Fungal biomass was scraped off the agar surface and DNA was extracted using the Qiagen DNeasy Plant 96 kit according to the manufacturer’s instructions (Qiagen N.V., Venlo, Limburg, Netherlands). DNA quality was checked by UV optical absorbance using an Epoch microplate spectrophotometer (BioTek Instruments, Inc., Winooski, VT, USA). Concentrations were measured using a Qubit^®^ fluorimeter and a QuantIt^™^ broad range assay kit (Invitrogen^™^, Thermofisher Scientific, Waltham, MA, USA).

#### 4.7.2. Fusarium Genus Primer Design

Two measures of *Fusarium* content were quantified in the seeds: total *Fusarium* content (genus level DNA) and the most common species, *F. verticillioides*, isolated from the Nampa ID trials. Q-PCR for *F. verticillioides* was handled as a separate analysis. Nicolaisen et al. [69] developed *Fusarium* species-specific primer pairs from within the tEF1α gene for 11 *Fusarium* species which are commonly found in cereals and other crops. During our study four species of *Fusarium* were repeatedly isolated from the seeds and identified. Three of these had specific qPCR primers developed by Nicolaisen et al. [69]; the fourth had not.

To identify regions of sequence conservation in translation elongation factor alpha gene among all *Fusarium* species of interest we aligned all the tEF1α sequences available in GenBank for 13 *Fusarium* species that are commonly found in crops in temperate regions. The species included in the alignment were *F. avenaceum*, *F. culmorum*, *F. equiseti*, *F. graminearum*, *F. langsethiae*, *F. poae*, *F. proliferatum*, *F. pseudograminearum*, *F. sporotrichioides*, *F. subglutinans*, *F. temperatum*, *F. tricinctum*, and *F. verticillioides*. The alignment of 504 sequences was done in CLCBio Main Workbench software (Qiagen Bioinformatics, Aarhus, Denmark). We were able to identify a region where an amplicon of between 50 and 100 bases could be produced for about 90% of the aligned sequences using an individual forward primer sequence (FsGns547F ACGCCTGGGTTCTTGACA) and a mixture of 3 similar reverse primer sequences (Rev1 FsGnsallR CGGTGACATAGTAGCGAGGAGT, Rev2 FsGnspoaeR GGTGACATAGTAGCGGGGAGT, and Rev3 FsGnstmprR TGACAGTGACATAGTAGCGAGGAG). The forward primer was used at a final concentration of 0.167 µM in the qPCR reaction and the reverse primers were used at 0.133 µM each. All primers have a predicted melting temperature (Tm) of roughly 62 °C.

#### 4.7.3. Quantitative PCR Assay

The amplification conditions and primer concentrations were tested in endpoint PCRs and analyzed on 5% high-resolution blend agarose gels (Lonza, Basel, Switzerland). Endpoint PCRs were done using MyTaq Red 2x master mix (Bioline, London, UK). Primer dimers which were formed at typical endpoint PCR primer concentrations were avoided by dilution of the primers. Only the expected products were made when *Fusarium* DNA was used as template and the selected primer concentrations were used. Some higher molecular weight material was sometimes produced especially when antagonist-treated seed DNA was used as template. We largely avoided this by keeping the extension times to a minimum and using a higher annealing temperature in the first 5 cycles, in a 2-step cycling program. All qPCRs were performed using SYBR Fast 2× Mix reagent (Bioline, London, UK) and reactions were carried out on an MJ Research Chromo4 (BioRad, Des Plaines, IL, USA) instrument. Once the cycling conditions were optimized for qPCR clean melting curves were obtained and standard curves were linear with from 10 ng down to 1 pg of *Fusarium* DNA from the four *Fusarium* species isolated during this study. The identities of the PCR products were checked by Sanger sequencing [70] and found to be as expected.

The thermal cycling program for the total *Fusarium* genus qPCR assay was as follows:

1. 95 °C3 min2. 95 °C5 s3. 68 °C8 s4. Go to 22×5. 95 °C5 s6. 67 °C8 s7. Go to 51×8. 95 °C5 s9. 66 °C18 s10. Plate read
11. Go to 834×12. Melting curve70 °C to 95 °C in 0.5 °C increments, 5 s per step.

The cycling program for the *F. verticillioides* primers was the same but with a 2 °C lower annealing/extension temperature.

The quantity of plant DNA in each seed DNA sample was measured by qPCR using the hor1F and hor2R plant-specific primers as described in Nicolaisen et al. [69]. Seed DNA from one test plot sample that gave the same DNA concentrations in both the Qubit^®^ and Epoch™ DNA assays was used to construct the standard curve for the plant DNA assay. As determined by Qubit^®^ measurement, 25ng of seed DNA was used in each qPCR for *Fusarium* assays.

Spiking excess pure fungal DNA into qPCRs containing seed DNA showed that there was some PCR inhibition associated with the seed DNA extracts. A correction factor was calculated by spiking two samples of seed DNA from each of the 8 sets of test plot samples with an excess of *F. verticillioides* DNA and comparing the calculated quantity to that obtained with the fungal DNA alone. This was done for both the Fusarium genus assay and the *F. verticillioides* assay.

### 4.8. Statistical Analyses

Propagule counts for *Fusarium* species in laboratory antagonism assays were non-parametric and were analyzed using the Kruskal-Wallis Wilcoxon rank score estimate in the NPAR1WAY procedure using SAS/STAT^®^ software (SAS^®^ 9.4, 2017, SAS Institute Inc., Cary, NC, USA) [71]. Mean comparisons were made using standard error as computed from mean score standard deviations.

All absorbance data from mycotoxin ELISA assays were transformed to concentrations using a logit-log function (Romer Labs, Inc. Newark, DE, USA) to generate a standard curve. Concentration data were then analyzed by analysis of variance and Fisher’s LSD and least squares mean comparisons using the GLM and MIXED procedures in SAS.

Results of the *Fusarium* genus qPCRs and *F. verticillioides* qPCRs, each measured on triplicate samples, were corrected for inhibition in each set of test plot samples and expressed as pg of total *Fusarium* or *F. verticillioides* DNA per ng of plant DNA. qPCR data analysis was performed using the web-based PCR Miner software package [72]. Threshold cycle values were standardized for conversion to picograms of *Fusarium* DNA per nanograms of seed DNA. These data were then analyzed using the Kruskal-Wallis Wilcoxon rank score estimate in the NPAR1WAY procedure in SAS. Mean comparisons were made using standard error as computed from mean score standard deviations.

## References

[1] Mlot C. (2009). Antibiotics in nature: Beyond biological warfare. Science.

[2] Czárán T.L., Hoekstra R.F., Pagie L. (2002). Chemical warfare between microbes promotes biodiversity. Proc. Natl. Acad. Sci. USA.

[3] Janzen D.H. (1977). Why fruits rot, seeds mold, and meat spoils. Am. Nat..

[4] Bérdy J. (2005). Bioactive microbial metabolites. J. Antibiot..

[5] Placinta C.M., D’mello J.P.F., Macdonald A.M.C. (1999). A review of worldwide contamination of cereal grains and animal feed with Fusarium mycotoxins. Anim. Feed Sci. Technol..

[6] Miedaner T., Bolduan C., Melchinger A.E. (2010). Aggressiveness and mycotoxin production of eight isolates each of Fusarium graminearum and Fusarium verticillioides for ear rot on susceptible and resistant early maize inbred lines. Eur. J. Plant Pathol..

[7] Stenglein S.A. (2009). Fusarium poae: A pathogen that needs more attention. J. Plant Pathol..

[8] National Grain and Feed Association (2011). FDA Mycotoxin Regulatory Guidance: A Guide for Grain Elevators, Feed Manufacturers, Grain Processors and Exporters. https://www.ngfa.org/wp-content/uploads/NGFAComplianceGuide-FDARegulatoryGuidanceforMycotoxins8-2011.pdf.

[9] United States Food and Drug Administration (2015). Mycotoxin Handbook. https://www.gipsa.usda.gov/fgis/handbook/MycotoxinHB/MycotoxinHandbook_2016-07-12.pdf.

[10] Baldwin T.T., Zitomer N.C., Mitchell T.R., Zimeri A.M., Bacon C.W., Riley R.T., Glenn A.E. (2014). Maize seedling blight induced by Fusarium verticillioides: Accumulation of Fumonisin B1 in leaves without colonization of the leaves. J. Agric. Food Chem..

[11] Covarelli L., Beccari G., Steed A., Nicholson P. (2012). Colonization of soft wheat following infection of the stem base by Fusarium culmorum and translocation of deoxynivalenol to the head. Plant Pathth..

[12] Desjardins A.E., Proctor R.H., Bai G., McCormick S., Shaner G.E., Buechley G., Hohn T.M. (1996). Reduced virulence of trichothecene-nonproducing mutants of Gibberella zeae in wheat field tests. Mol. Plant Microbe Interact..

[13] Harris L.J., Desjardins A.E., Plattner R.D., Nicholson P., Butler G., Young J.C., Weston G., Proctor R.H., Hohn T.M. (1999). Possible role of trichothecene mycotoxins in virulence of Fusarium graminearum on maize. Plant Dis..

[14] Quesada-Ocampo L.M., Al-Haddad J., Scruggs A.C., Buell C.R., Trail F. (2016). Susceptibility of maize to stalk rot caused by *Fusarium graminearum* deoxynivalenol and zearalenone mutants. Phytopathology.

[15] Gu Q., Tahir H.A.S., Zhang H., Huang H., Ji T., Sun X., Wu L., Wu H., Gao X. (2017). Involvement of FvSet1 in fumonisin b1 biosynthesis, vegetative growth, fungal virulence, and environmental stress responses in *Fusarium verticillioides*. Toxins.

[16] Ridenour J.B., Bluhm B.H. (2017). The novel fungal-specific gene FUG1 has a role in pathogenicity and fumonisin biosynthesis in *Fusarium verticillioides*. Mol. Plant Pathol..

[17] Ridenour J.B., Smith J.E., Bluhm B.H. (2016). The HAP complex governs fumonisin biosynthesis and maize kernel pathogenesis in *Fusarium verticillioides*. J. Food Prot..

[18] Desmond O.J., Manners J.M., Stephens A.E., Maclean D.J., Schenk P.M., Gardiner D.M., Munn A.L., Kazan K. (2008). The Fusarium mycotoxin deoxynivalenol elicits hydrogen peroxide production, programmed cell death and defense responses in wheat. Mol. Plant Pathol..

[19] Leslie J.F., Plattner R.D., Desjardins A.E., Klittich C.J. (1992). Fumonisin B1 production by strains from different mating populations of *Gibberella fujikuroi* (*Fusarium* section *Liseola*). Phytopathology.

[20] Proctor R.H., Plattner R.D., Desjardins A.E., Busman M., Butchko R.A. (2006). Fumonisin production in the maize pathogen *Fusarium verticillioides*: Genetic basis of naturally occurring chemical variation. J. Agric. Food Chem..

[21] Munkvold G.P., Xu X., Bailey J.A., Cooke M. (2003). Epidemiology of Fusarium diseases and their mycotoxins in maize ears. Epidemiology of Mycotoxin Producing Fungi.

[22] Estrada A.E.R., Jonkers W., Kistler H.C., May G. (2012). Interactions between Fusarium verticillioides, Ustilago maydis, and Zea mays: An endophyte, a pathogen, and their shared plant host. Fungal Genet. Biol..

[23] Lee K., Pan J.J., May G. (2009). Endophytic *Fusarium verticillioides* reduces disease severity caused by Ustilago maydis on maize. FEMS Microbiol. Lett..

[24] Pal K.K., Tilak K.V.B.R., Saxcna A.K., Dey R., Singh C.S. (2001). Suppression of maize root diseases caused by Macrophomina phaseolina, *Fusarium moniliforme* and Fusarium graminearum by plant growth promoting rhizobacteria. Microbiol. Res..

[25] Bacon C.W., Yates I.E., Hinton D.M., Meredith F. (2001). Biological control of *Fusarium moniliforme* in maize. Environ. Health Perspect..

[26] Bacon C.W., Hinton D.M., Porter J.K., Glenn A.E., Kuldau G. (2004). Fusaric acid, a Fusarium verticillioides metabolite, antagonistic to the endophytic biocontrol bacterium Bacillus mojavensis. Can. J. Bot..

[27] Mousa W.K., Shearer C.R., Limay-Rios V., Zhou T., Raizada M.N. (2015). Bacterial endophytes from wild maize suppress Fusarium graminearum in modern maize and inhibit mycotoxin accumulation. Front. Plant Sci..

[28] Pereira P., Nesci A., Castillo C., Etcheverry M. (2010). Impact of bacterial biological control agents on fumonisin B 1 content and *Fusarium verticillioides* infection of field-grown maize. Biol. Control.

[29] Velluti A., Marín S., Gonzalez R.J., Ramos A., Sanchis V. (2001). Fumonisin B1, zearalenone and deoxynivalenol production by *Fusarium moniliforme*, *F proliferatum* and *F graminearum* in mixed cultures on irradiated maize kernels. J. Sci. Food Agric..

[30] Keyser Z., Vismer H.F., Klaasen J.A., Snijman P.W., Marasas W.F.O. (1999). The antifungal effect of fumonisin B~ 1 on *Fusarium* and other fungal species. S. Afr. J. Sci..

[31] Bacon C.W., Hinton D.M., Hinton A. (2006). Growth-inhibiting effects of concentrations of fusaric acid on the growth of *Bacillus mojavensis* and other biocontrol *Bacillus* species. J. Appl. Microbiol..

[32] Yates I.E., Meredith F., Smart W., Bacon C.W., Jaworski A.J. (1999). *Trichoderma viride* suppresses fumonisin B1 production by *Fusarium moniliforme*. J. Food Prot..

[33] Chatterjee S., Kuang Y., Splivallo R., Chatterjee P., Karlovsky P. (2016). Interactions among filamentous fungi *Aspergillus niger*, *Fusarium verticillioides* and *Clonostachys rosea*: Fungal biomass, diversity of secreted metabolites and fumonisin production. BMC Microbiol..

[34] Hebbar K.P., Davey A.G., Dart P.J. (1992). Rhizobacteria of maize antagonistic to *Fusarium moniliforme*, a soil-borne fungal pathogen: Isolation and identification. Soil Biol. Biochem..

[35] Reid L.M., Nicol R.W., Ouellet T., Savard M., Miller J.D., Young J.C., Stewart D.W., Schaafsma A.W. (1999). Interaction of *Fusarium graminearum* and *F. moniliforme* in maize ears: Disease progress, fungal biomass, and mycotoxin accumulation. Phytopathology.

[36] Bacon C.W., Hinton D.M. (1996). Symptomless endophytic colonization of maize by *Fusarium moniliforme*. Can. J. Bot..

[37] Foley D.C. (1962). Systemic infection of corn by *Fusarium moniliforme*. Phytopathology.

[38] Picot A., Barreau C., Pinson-Gadais L., Piraux F., Caron D., Lannou C., Richard-Forget F. (2011). The dent stage of maize kernels is the most conducive for fumonisin biosynthesis under field conditions. Appl. Environ. Microbiol..

[39] Velluti A., Marın S., Bettucci L., Ramos A.J., Sanchis V. (2000). The effect of fungal competition on colonization of maize grain by *Fusarium moniliforme*, *F. proliferatum* and *F. graminearum* and on fumonisin B 1 and zearalenone formation. Int. J. Food Microbiol..

[40] Idaho State Dept of Agriculture (2016). Agricultural Facts. http://www.agri.idaho.gov/agri/Categories/Marketing/Documents/2016AgStats.pdf.

[41] Datnoff L.E., Elmer W.H., Huber D.M. (2007). Mineral Nutrition and Plant Disease.

[42] Jensen C.A., Olshausen B.A. (1901). Soil Survey of Boise Area, Idaho.

[43] Headrick J.M., Pataky J.K., Juvik J.A. (1990). Relationships among carbohydrate content of kernels, condition of silks after pollination, and the response of sweet corn inbred lines to infection of kernels by *Fusarium moniliforme*. Phytopathology.

[44] Wilson D.O., Mohan S.K. (1998). Unique seed quality problems of sh2 sweet corn. Seed Technol..

[45] Duncan K.E., Howard R.J. (2010). Biology of maize kernel infection by *Fusarium verticillioides*. Mol. Plant Microbe Interact..

[46] Wilke A.L., Bronson C.R., Tomas A., Munkvold G.P. (2007). Seed transmission of *Fusarium verticillioides* in maize plants grown under three different temperature regimes. Plant Dis..

[47] Bacon C.W., Hinton D.M. (2002). Endophytic and biological control potential of Bacillus mojavensis and related species. Biol. Control.

[48] Ridout M.E., Newcombe G., Godfrey B. (2016). First report of *Fusarium temperatum* in biseased sweet corn ears in the western United States. Plant Dis..

[49] Aytoun R.S. (1956). The effects of griseofulvin on certain phytopathogenic fungi. Ann. Bot..

[50] Brian P.W. (1954). The use of antibiotics for control of plant diseases caused by bacteria and fungi. J. Appl. Bacteriol..

[51] Brian P.W., Wright J.M., Stubbs J., Way A.M. (1951). Uptake of antibiotic metabolites of soil micro-organisms by plants. Nature.

[52] Crowdy S.H., Grovem J.F., Hemming H.G., Robinson K.C. (1956). The translocation of antibiotics in higher plants: II. The movement of griseofulvin in broad bean and tomato. J. Exp. Bot..

[53] Stokes A. (1954). Uptake and translocation of griseofulvin by wheat seedlings. Plant Soil.

[54] Heleno S.A., Stojković D., Barros L., Glamočlija J., Soković M., Martins A., Queiroz M.J.R., Ferreira I.C. (2013). A comparative study of chemical composition, antioxidant and antimicrobial properties of *Morchella esculenta* (L.) Pers. from Portugal and Serbia. Food Res. Internat..

[55] Tietel Z., Masaphy S. (2017). True morels (*Morchella*)—Nutritional and phytochemical composition, health benefits and flavor: A review. Crit. Rev. Food Sci. Nutr..

[56] Turkoglu A., Kivrak I., Mercan N., Duru M.E., Gezer K., Turkoglu H. (2006). Antioxidant and antimicrobial activities of *Morchella conica* Pers. Afr. J. Biotechnol..

[57] Mohan S.K., Wilson D.O. (1990). Fungi associated with seed and seedling blight of shrunken2 sweet corn in Idaho. Phytopathology.

[58] Sutton J.C., Baliko W., Funnell H.S. (1976). Evidence for translocation of zearalenone in corn plants colonized by *Fusarium graminearum*. Can. J. Plant Sci..

[59] Mihuta-Grimm L., Forster R.L. (1989). Scab of wheat and barley in southern Idaho and evaluation of seed treatments for eradication of *Fusarium* spp.. Plant Dis..

[60] Baird R.E., Huber D.M., Mullinix B.G. (1995). The mycobiota from seeds of shrunken-2 (sh2) sweet corn. Mycopathologia.

[61] Wilson D.O., Mohan S.K., Knott E.A., Shafii B. (1993). Evaluation of fungicide seed treatments for Shrunken-2 (“Supersweet”) sweet corn. Plant Dis..

[62] Miller J.D. (2001). Factors that affect the occurrence of fumonisin. Environ. Health Perspect..

[63] Picot A., Barreau C., Pinson-Gadais L., Caron D., Lannou C., Richard-Forget F. (2010). Factors of the *Fusarium verticillioides*-maize environment modulating fumonisin production. Crit. Rev. Microbiol..

[64] Campanile G., Ruscelli A., Luisi N. (2007). Antagonistic activity of endophytic fungi towards *Diplodia corticola* assessed by *in vitro* and *in planta* tests. Eur. J. Plant Pathol..

[65] De Capdeville G., Wilson C.L., Beer S.V., Aist J.R. (2002). Alternative disease control agents induce resistance to blue mold in harvested ‘Red Delicious’ apple fruit. Phytopathology.

[66] Martín J.A., Macaya-Sanz D., Witzell J. (2015). Strong *in vitro* antagonism by elm xylem endophytes is not accompanied by temporally stable in planta protection against a vascular pathogen under field conditions. Eur. J. Plant Pathol..

[67] Ridout M., Newcombe G. (2016). Disease suppression in winter wheat from novel symbiosis with forest fungi. Fungal Ecol..

[68] European Commission Joint Research Centre, Institute for Health and Consumer Protection (2005). Event-Specific Method for the Quantification of Maize Line NK603 Using Real-Time PCR. http://gmo-crl.jrc.ec.europa.eu/summaries/NK603-WEB-ProtocolValidation.pdf.

[69] Nicolaisen M., Supronienė S., Nielsen L.K., Lazzaro I., Spliid N.H., Justesen A.F. (2009). Real-time PCR for quantification of eleven individual *Fusarium* species in cereals. J. Microbiol. Meth..

[70] Sanger F., Nicklen S., Coulson A.R. (1977). DNA sequencing with chain-terminating inhibitors. Proc. Natl. Acad. Sci. USA.

[71] SAS Institute Inc. (2017). SAS^®^ 9.4.

[72] Zhao S., Fernald R.D. (2005). Comprehensive algorithm for quantitative real-time polymerase chain reaction. J. Comput. Biol..

